# Oral manifestation of Waardenburg syndrome: a case report and review of the literature

**DOI:** 10.1259/bjrcr.20200071

**Published:** 2020-06-24

**Authors:** Rohan Jagtap, Ambika Srivastava, Aniket Jadhav, Swati Gupta

**Affiliations:** 1 Department of Care planning and Restorative Sciences, University of Mississippi Medical Center School of Dentistry, Jackson, Mississippi; 2 Department of Oral and Maxillofacial Radiology, Virginia Commonwealth University School of Dentistry, Richmond, Virginia; 3 Department of Periodontology, University of Florida College of Dentistry, Gainesville, Florida

## Abstract

Waardenburg syndrome is a rare autosomal dominant genetic disorder of neural crest cell migration. It is characterized by congenital sensorineural hearing loss, heterochromia iridis, depigmentation of hair and skin, and increased intercanthal distance. It is subdivided into four subtypes with I and II being most common. These subtypes are categorized based on genetic mutations. Although medical literature has well documented this syndrome, dental and radiographical findings have been rarely presented. In this case report and literature review, we have presented and discussed oral as well as head and neck radiology findings of a 20-year-old girl with Waardenburg syndrome.

## Introduction

Waardenburg syndrome (WS) is a rare hereditary disorder described first by Petrus Johannes Waardenburg in 1951. The characteristic clinical findings include sensorineural hearing loss, increased intercanthal distance, heterochromia iridis, pigmentary abnormalities of hair and skin along with dental findings of agenesis, cleft lip and/ palate and tooth malformations.^1^

Based on the genetic mutations, six specific genes are involved with phenotypic diversity PAX3, SOX10, MITF, SNAI2, EDN3, and EDNRB. It should be noted that all forms of WS are variable, even within families, making it difficult to predict the severity of WS. WS is diagnosed through the presence of two major or one major and two minor criteria (Table 1).^2^

**Table 1.** Diagnostic criteria for Waardenburg syndrome.

Major criteria	Minor criteria
Congenital sensorineural hearing loss	Congenital leucoderma
Pigmentary disturbances of iris	Synophrys or medial eyebrow flare
Hair hypopigmentation	Broad and high nasal root
Dystopia canthorum	Hypoplasia of alae nasi
Affected first degree relative	Premature graying of hair (before age 30)

There are four different types of WS with Type I and Type II being the most common.^2,3^

- Type I: Wide space present between inner canthus; caused by loss of function mutations in PAX3 gene. 20% of cases have hearing impairment.
- Type II: 50% of cases have hearing impairment or deaf. Wide space between inner canthus is not noted. 15% are heterozygous for mutations in microphthalmia-associated transcription factor (MITF) gene.
- Type III: Limb abnormality; extreme presentation of type I. Also known as Klein-WS.
- Type IV: Most cases have Hirschsprung disease, caused by mutations in genes for endothelin-3 or one of its receptors EDNRB. Also known as Shah-WS.

The varying symptoms and clinical findings in each of the types could be attributed to the different genes involved.^4^ Generally, the symptoms include very pale or blue eye, heterochromia or eyes with iris having two different colors, forelock of white hair or premature graying, lateral displacement of medial canthi along with dystopia of lacrimal puncta and blepharophimosis, prominent broad nasal root, and moderate-to-profound hearing impairment.^4^ Currently, there is no cure for this syndrome. Although there is sufficient medical literature available on this syndrome, there are limited studies discussing the dental clinical and radiographical findings.

We report a case of 20-year-old girl with WS, who presented with unique clinical and radiological findings.

## Case presentation

A 20-year-old girl presented to her orthodontist with the chief complaint of pain in the jaws, that had been troubling her since few years and had gotten worse. The patient reported that pain had been interfering with her masticatory function. The practitioner referred the patient to the University of Florida College of Dentistry (UFCD) for evaluation of temporomandibular joint (TMJ) disorders. Patient’s medical records showed that she had WS which was in consistence with her symptoms of deafness, neurological disturbances (incoordination and neuropathy), and developmental delay. Other clinical findings were increased intercanthal distance, heterochromia (eyes with different color), premature gray hair, and broad nasal root. Patient presented with unilateral hearing loss with limited hearing in the left ear. This was unusual as bilateral hearing loss has been mentioned in most patients with WS.

Intraoral examination revealed an increased overjet, normal mouth opening of 35 mm with bilateral tender masseter muscle. Patient reported that she had never experienced trismus, dysphagia, paresthesia, or swelling/ intraoral drainage.

**Figure 1.**
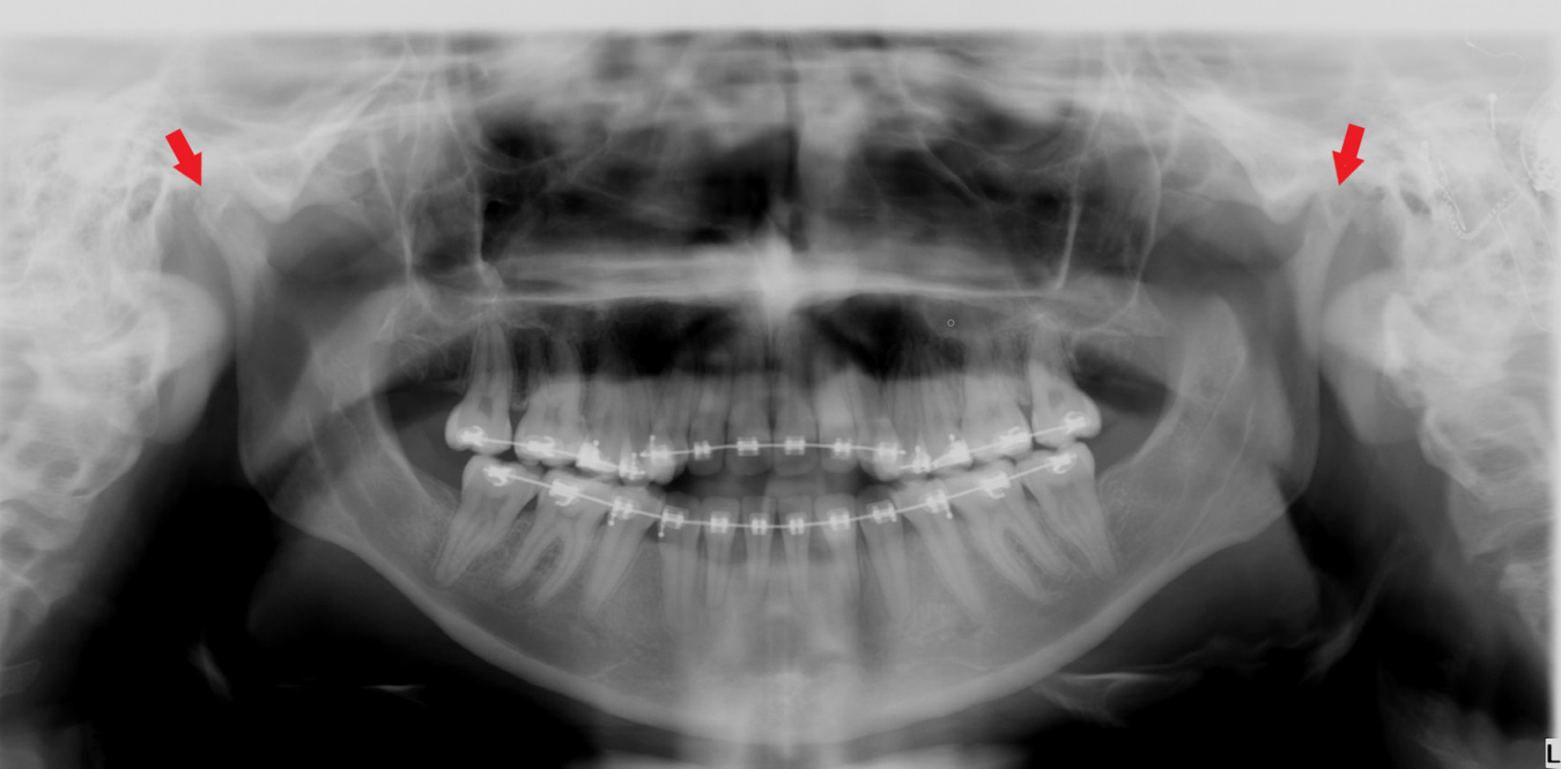
A panoramic radiograph exhibits condylar hypoplasia bilaterally (red arrows) and anterior apertognathia.

**Figure 2.**
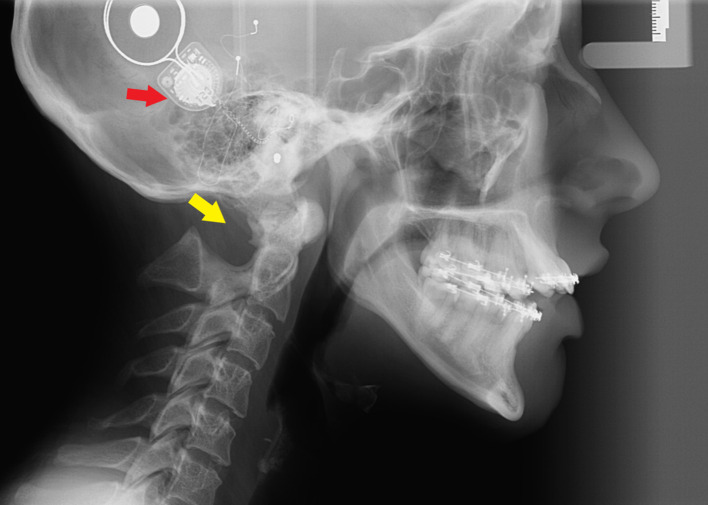
A lateral cephalogram exhibits cochlear implant associated with the left ear (red arrow), posterior arch aplasia of C1 (yellow arrow), prognathic chin, and increased overjet & open bite.

A pantomograph and cephalogram were made for the diagnostic purpose. The scan revealed anterior apertognathia, small-sized mandibular condyles, and posterior arch aplasia of C1 (Figures 1 and 2). There is a discrepancy in maxilla and mandible morphology which causes premature occlusion in posterior teeth that possibly results in apertognathia and increased overjet.

**Figure 3.**
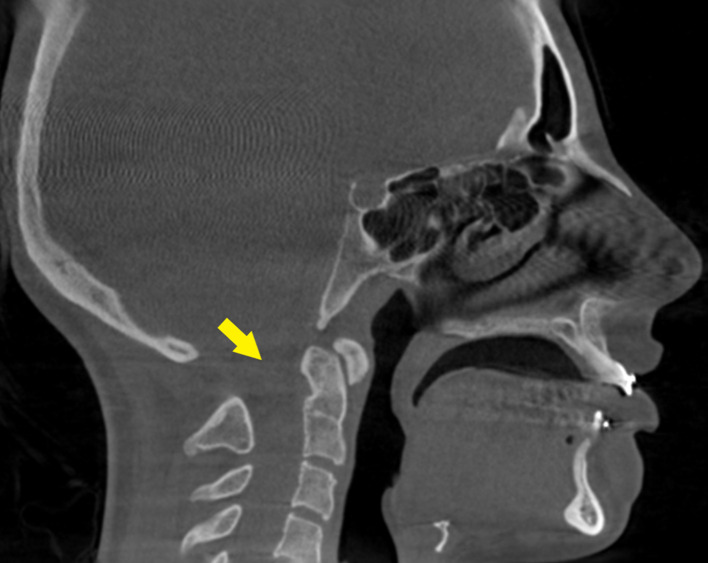
Parasagittal CBCT view depict posterior arch agenesis of C1 (yellow arrow) and cochlear implant associated with the left ear. There is a thinning of cortices in the mandibular anterior region.

**Figure 4.**
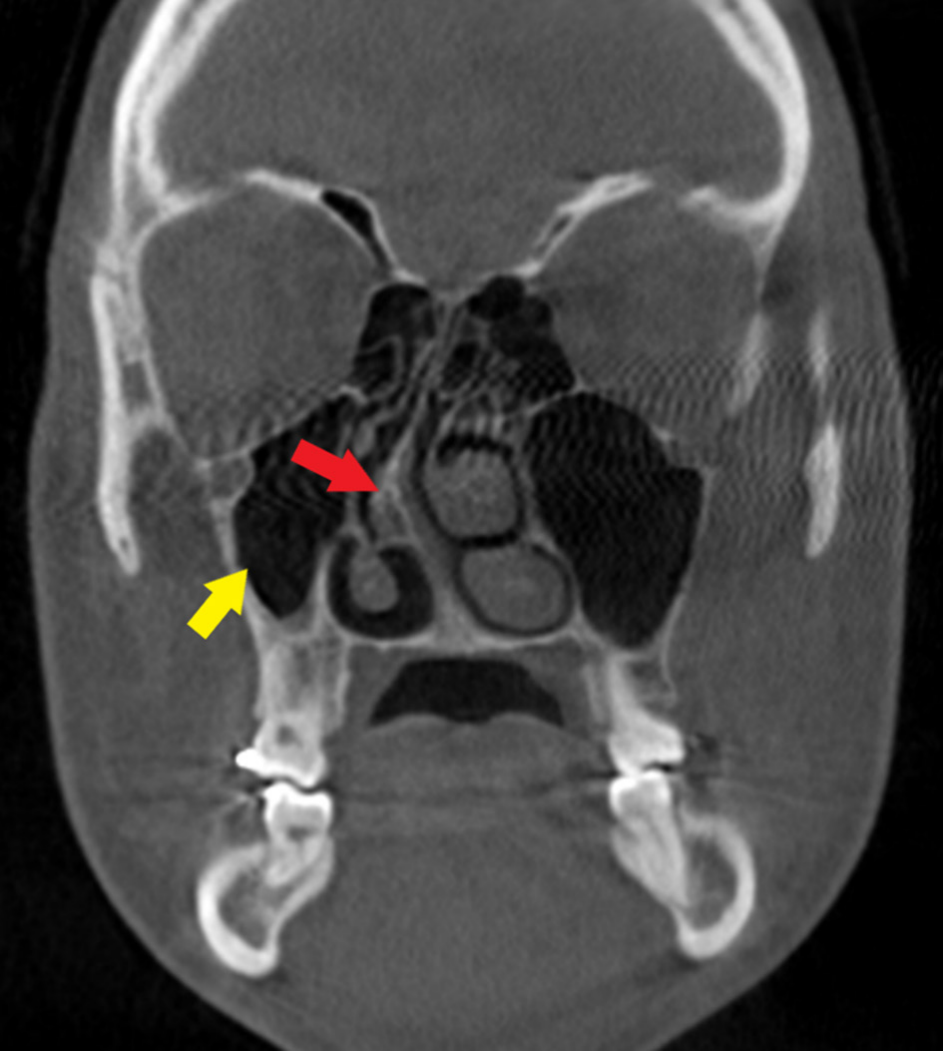
Reconstructed coronal view of CBCT shows right maxillary sinus hypoplasia (yellow arrow) and marked deviation of nasal septum to right side-with septal spur on the right (red arrow).

**Figure 5.**
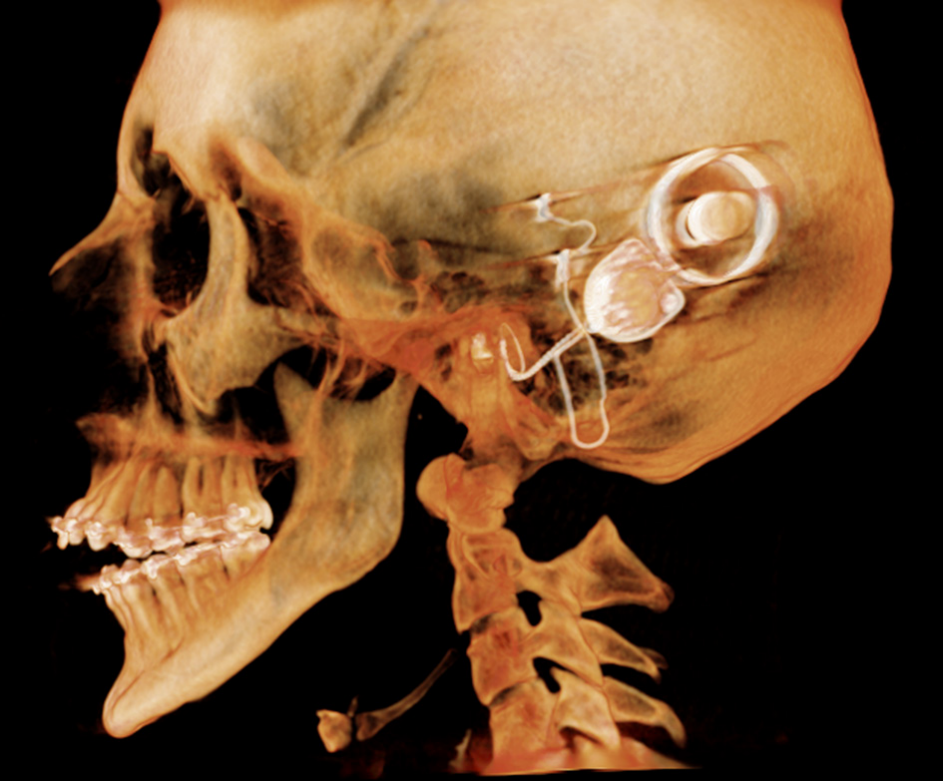
Sagittal view of 3D reconstruction.

To evaluate maxillofacial region, the clinician performed a cone-beam CT (CBCT) scan. The department of oral and maxillofacial radiology at the University of Florida College of Dentistry received a referral from clinician for radiographical interpretation of CBCT with emphasis on temporomandibular joints review. The CBCT volume extended from the level of frontal bone to C5 vertebrae. The CBCT showed malocclusion such as marked overjet and anterior apertognathia, right maxillary sinus hypoplasia and condylar hypoplasia bilaterally, posterior arch aplasia of C1 as seen in cephalogram, severe nasal septum deviation to the right side, and cochlear implant associated with the left ear (Figures 3–5). One of the important radiographical findings in Waardenburg syndrome patient is posterior arch agenesis. It is a rare finding; patients are mostly asymptomatic but may show some symptoms such as neck pain due to neurological deficits. Prevalence of posterior arch agenesis is rare and reported to be 0.15%.

## Discussion

The patient in the present study presented with the following findings:

Medical: Bilateral sensorineural hearing loss, bilateral blue iris, musculoskeletal abnormalities, cochlear implant associated with the left ear.

Facial and dental findings: Increased intercanthal distance, increased overjet, anterior apertognathia, and posterior arch agenesis of C1.

Similar medical findings have been reported by previous case reports.^2,5^ The differential diagnosis for this syndrome are Vogt-Koyanagi-Harada syndrome, oral-facial-digital syndrome, Tietz syndrome, and ocular albinism with sensorineural deafness.^6^ The craniofacial features of orofacial digital syndrome include frontal bossing, facial asymmetry, aplasia of alar cartilage, ocular hypertelorism, strabismus, downslanting palpebral fissures, broadened nasal bridge/root with oral manifestations such as cleft lip/palate, high-arched palate, ankyloglossia, oligodontia, supernumerary teeth, hyperplastic frenula, and micrognathia. Malformations of the hands such as syndactyly, brachydactyly, and clinodactyly are more common than those of the feet.^7^ No specific dental findings have been reported associated with the other three syndromes. These signs helped us to draw a definitive diagnosis of WS.

WS, a rare disease, is characterized by clinical manifestations of oculocutaneous anomalous pigmentation, deafness of varying degree, dystopia canthorum, and broad nasal root.^8^ The estimated prevalence of WS is 1:42,000, with the highest prevalence seen among Kenyan Africans. In case of non-dental findings, WS accounts for 1.43% of congenital deafness.^2,3^ There is significant amount of literature reporting the medical findings of WS, but hardly any on dental findings.

The first study reporting dental findings in WS reported dental agenesis of the lower lateral incisors in patients.^1^ Dental agenesis has been suggested to be more prevalent in permanent dentition as compared to primary dentition.^9^ A case report on WS reported dental findings of missing lateral incisor, fused lateral incisor, and canine as well as increased spacing between teeth.^7^ Multiple agenesis has been reported to be in 0.25% of the population affected with this disorder.^9^ Another study performed an extensive dermatological, ophthalmological, otorhinolaryngological, and orofacial analysis in 29 family members to report inheritance pattern and clinical features in WS patients. Dental anomalies such as dental agenesis, conical teeth, taurodontism, and oligodontia were the common findings in the study. Upper lateral incisors were the most common missing teeth (12.19%). A frequency of 9.75% is seen in missing lower lateral incisors, lower central incisors, upper and lower second premolars, second molars and third molars. Canines were mostly conical in (42.85%), followed by the lower incisors and canines, both with a frequency of 28.58%. This was found to be in contradiction to the other studies, which usually report upper lateral incisors to be conical in shape.^1^ Oligodontia, malformed conical teeth, can pose not only esthetic but functional problems for the patients. An early diagnosis of the syndrome, by dental clinician or medical professional, can help to develop a protocol to improve the quality of life of the patients.

In the present study, CBCT was done to determine radiological findings associated with the WS. The CBCT and cephalogram showed marked overjet and anterior apertognathia, right maxillary sinus and condylar hypoplasia, posterior arch aplasia of C1, and severe nasal septum deviation to the right side. As per our knowledge, no other case report has discussed CBCT findings. It could be due to financial constraints, limited resources in the facility or patient’s personal reasons. However, these radiological findings could be used to diagnose WS.

## Conclusion

WS is one of the rare syndromes associated with genetic mutations and presents with characteristic medical and dental findings. We have reported certain oral and radiological findings associated with the syndrome, thereby hoping to broaden knowledge about the varying findings in this disorder. More research with larger sample size is needed to understand the dental findings and develop a treatment protocol to address the same for oral rehabilitation.

## Learning points

1. Waardenburg syndrome (WS) is a rare autosomal dominant genetic disorder.
2. Based on different features, there are various subtypes of WS; Type 1 and Type 2 being most common.
3. WS is characterized by medical findings of bilateral sensorineural hearing loss, bilateral blue iris, musculoskeletal abnormalities, and cochlear implant associated with the left ear. The facial and dental findings observed are increased intercanthal distance, increased overjet, anterior apertognathia, and posterior arch agenesis of C1.
4. Currently, there is no cure for this syndrome. Radiographical interpretation and recognition of multiple abnormalities associated with this syndrome is important.
5. A multidisciplinary approach involving both dentists and physicians plays a key role in management of WS.

## References

[1] Solia-Nasser L, de Aquino SN, Paranaiba LMR, Gomes A, dos-Santos-Neto P, Coletta RD, et al Waardenburg syndrome type I: dental phenotypes and genetic analysis of an extended family. Med Oral 2016; 21: e321: e321–7. doi: 10.4317/medoral.20789 PMC4867205 27031059

[2] Rawlani S, Ramtake R, Dhabarde A, Rawlani S Waardenburg syndrome: a rare case. Oman journal of ophthalmology 2018; 11. 10.4103/ojo.OJO_51_2014 PMC5991067 29930451

[3] Read AP, Newton VE Waardenburg syndrome. J Med Genet 1997; 34: 656–65. doi: 10.1136/jmg.34.8.656 9279758 PMC1051028

[4] Kumar S, Rao K Waardenburg syndrome: a rare genetic disorder, a report of two cases. Indian journal of human genetics 2012; 18. 10.4103/0971-6866.100804 PMC3491306 23162308

[5] Laababsi R “Waardenburg Syndrome: A Rare Cause of Sensorineural Hearing Loss in Infancy”. EC Dental Science 2018; 10: 1680–4 17.

[6] Waardenburg syndrome.Retrieved from Available from: https://radiopaedia.org/articles/waardenburg-syndrome-1?lang=us.

[7] Tuli A, Singh A, Sachdev V, Kumar A Physical and dental manifestations of oral-facial-digital syndrome type I. J Indian Soc Pedod Prev Dent 2011; 29, :: 83–6 Suppl S1. doi: 10.4103/0970-4388.90750 22169845

[8] Eigelshoven S, Kameda G, Kortüm A-K, Hübsch S, Angerstein W, Singh P, et al Waardenburg syndrome type I with heterochromia iridis and circumscribed hypopigmentation of the skin. Pediatr Dermatol 2009; 26: 759–61. doi: 10.1111/j.1525-1470.2009.01033.x 20199465

[9] Moussa MM, Hamed RS Waardenburg syndrome type I with dental anomaly: case report and literature review. Int J Case Rep Images 2019;10:100990Z01MM 2019;.

